# Case Report: Culture-Dependent Postures in Japanese Patients With Schizophrenia

**DOI:** 10.3389/fpsyt.2021.686817

**Published:** 2021-08-05

**Authors:** Akihiro Koreki, Teruki Koizumi, Kamiyu Ogyu, Mitsumoto Onaya

**Affiliations:** Department of Psychiatry, National Hospital Organization Shimofusa Psychiatric Medical Center, Chiba, Japan

**Keywords:** schizophrenia, culture dependent, fear, guilty, Japan, social cognition

## Abstract

Cross-cultural understanding of psychiatric symptoms is important in the current globalised society. Lack of knowledge regarding culture-dependent manifestations of psychiatric illnesses may lead to misjudgement by clinicians, resulting in inappropriate treatment. We present the cases of two patients with schizophrenia who showed Japanese-culture-dependent postures (*seiza* and *dogeza*). *Seiza* is a Japanese style of formal floor sitting. *Dogeza* includes bowing and touching the forehead to the floor while sitting in a kneeling position. When patients with schizophrenia perform these postures in a clinical setting, clinicians receive plenty of information regarding the patients' clinical states, including schizophrenia-related fear/tension, accusatory auditory verbal hallucinations, and pathological guilt.

## Introduction

Despite an increasing amount of research (1), no biomarker for schizophrenia has been identified, and clinical assessment based on verbal examination and behavioural observation remains essential to gauge the psychiatric condition of patients with schizophrenia. In the current scenario of a global society, lack of knowledge regarding culture-dependent behaviours may lead to misjudgement by clinicians, resulting in inappropriate treatment. Therefore, there is a pressing need to understand culture-dependent behaviours, especially if they reflect schizophrenic symptoms such as social cognition impairment. While cultural differences in the psychotic experiences of patients with schizophrenia have been investigated (2, 3), to the best of our knowledge, no study has reported culture-dependent postures as an observable sign of schizophrenia.

**Figure 1:**
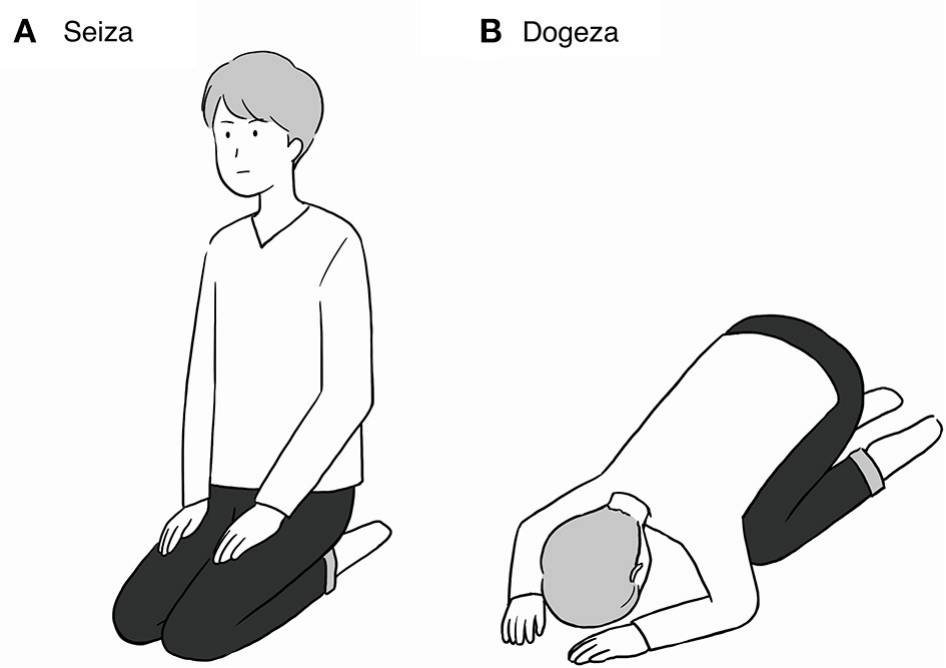
Cartoons are also a part of Japanese culture. **(A)** *Seiza* is a style of Japanese formal sitting that involves kneeling on the floor. **(B)** *Dogeza* is a form of bowing while sitting in a kneeling position with the forehead touching the floor.

In our clinical experience at our psychiatric hospital, we observed that some patients with schizophrenia behave inappropriately formally toward medical personnel such as doctors. For example, they immediately stand up when a doctor enters their room, bow deeply, and use formal language when talking to medical personnel even when it is not required. Moreover, they kneel on their bed in the “*seiza*” position (Figure 1A). *Seiza* is a Japanese style of formal floor sitting (4). This custom is still followed in Japanese society despite the Westernisation of lifestyles and chair sitting becoming more common. Patients with schizophrenia sometimes perform *seiza* in clinical practise, which is bizarre and inappropriately and excessively formal.

In addition to *seiza*, the “*dogeza*” posture is occasionally observed (5). It includes bowing and touching the forehead to the floor while sitting in a kneeling position (Figure 1B). *Dogeza* is performed to convey the following two meanings (6, 7): (1) an excessively formal (or comedic) style of entreating someone and (2) expressing a deeply sincere apology. *Dogeza* is rarely performed in daily life for either purpose and is often seen in fictional works, such as comedies and dramas. Therefore, it gives an even more bizarre impression than *seiza* when encountered in clinical practise.

There are several Japanese-culture-related concepts. “Bowing” is the act (rather than posture) of lowering the head, and Japanese people usually greet each other with a light bow instead of a handshake (8). A deeper and longer bow is usually performed to express great respect or apology. Bowing may be performed in both the standing and sitting positions. “Prostration,” which may include bowing, sitting, and lying stretched out, has religious connotations, and the posture varies depending on the religion (9, 10). It is performed to express submissiveness or worship to a supreme being and may also be performed in front of rulers or social superiors (8). In contrast to *seiza*, which is considered formal, “*agura*” is a style of cross-legged floor sitting that is considered casual in Japanese society (4).

These postures and behaviours have a complicated history, terminological relationship, and association with religions, and many theories about them exist (4–10). However, these culture-dependent postures have not yet been discussed in relation to the field of psychiatry, although *seiza*-induced entrapment neuropathy (11) and difficulty in s*eiza* sitting due to knee problems (12) have been reported in the fields of neurology and orthopaedics, respectively. Therefore, we aimed to focus on the clinical meanings of these postures in the field of psychiatry. Performance of these postures by patients with schizophrenia in the clinical setting provides clinicians with plenty of information regarding the patients' clinical states. Here, we report on two patients with schizophrenia who showed these Japanese-culture-dependent postures, and discuss the clinical implications of these postures. We obtained written informed consent for reporting these cases from both patients.

## Case Description

### Case 1

A 45-year-old man with schizophrenia diagnosed based on the Diagnostic and Statistical Manual of Mental Disorders, fifth edition criteria (DSM-5) was admitted to our hospital under the care of AK due to psychotic symptoms including verbal hallucinations, delusions of reference, cenesthesic hallucinations, fear, tension, anxiety, impulsivity, and tongue self-injury. He was not religious, had no comorbidities, and had an older brother who had schizophrenia. The onset of his symptoms was at age 20, and he had a history of multiple admissions (more than 10 times) due to symptom recurrence, resulting in progression of cognitive impairment and treatment-resistance even for positive symptoms. Although he had no specific fear or anxiety, he experienced schizophrenia-related general fear and anxiety. During hospitalisation, he underwent modified electroconvulsive therapy and received various antipsychotic drugs (including depot injections). Despite this, his symptoms fluctuated. However, his social skills remained relatively good and he could plan and manage his daily life after discharge. During this unstable clinical course, we noticed that he sometimes performed *seiza* on his bed when he met medical personnel such as doctors. Occasionally, he performed *dogeza* as well as *seiza* when he asked for something, such as cigarettes and drugs. Although the request itself was reasonable, considering the situation, these postures seemed inappropriate, bizarre, and stereotyped. More importantly, he performed these postures and deep bowing during periods of symptom deterioration, i.e., when his symptoms were unstable. On being asked the reason for the postures, he answered that he felt sorry for the doctor and did not provide any further details and that he performed *dogeza* to indicate an entreaty rather than an apology. While he denied any association with hallucination or delusion, he accepted an association with fear, tension, and anxiety, implying that these postures were signs of symptom deterioration. At the time of discharge, he was being treated with 150 mg paliperidone palmitate and maintenance modified electroconvulsive therapy. His scores on the positive, negative and general subscales of the Positive and Negative Syndrome Scale (PANSS) improved from 30, 28, and 60 on admission to 15, 27, and 35 at the time of discharge, respectively.

### Case 2

Our other patient was a 46-year-old woman with schizophrenia diagnosed based on the DSM-5. She had no significant past medical history or family history of schizophrenia. The onset of her symptoms was at age 36, and she had a history of four admissions due to severe psychosis including auditory verbal hallucinations, such as “I will kill you” and “I hope you die,” delusions of reference, guilt, and pregnancy, and moderate negative symptoms. During initial hospitalisation, she frequently performed *dogeza* in the corridor and at the bedside. On being asked the reason for this, she answered that she needed to apologise because her auditory hallucinations led her to being accused of breaking things and stealing money (these were clearly her delusions), for which she felt guilty. Furthermore, she showed a posture of “*harakiri*” (suicide by cutting one's stomach to express apology) and asked medical personnel to kill her. During one admission when AK was in charge of her treatment, she was administered clozapine (up to 375 mg) and her symptoms gradually improved. *Dogeza* frequency decreased with symptom improvement until it finally disappeared. Her scores on the positive, negative, and general subscales of the PANSS improved from 33, 31, and 89 on admission to 14, 26, and 47 after treatment, respectively.

## Discussion

Of the two patients whose cases are presented here, one showed *seiza* and the entreaty type of *dogeza* along with schizophrenic symptoms of abnormal fear and tension, and the other showed the apology type of *dogeza* along with accusatory auditory verbal hallucinations. These postures are rooted in Japanese culture, and lack of knowledge regarding them may lead clinicians from foreign countries to misunderstand and misinterpret them as catatonic features. However, although these postures seem excessive and contextually bizarre, unlike catatonic features, they serve some purpose such as indicating formality or apology. In addition, in Patient 1, *seiza* and *dogeza* reflected fear and tension and could be considered as a warning sign of symptom deterioration. In Patient 2, *dogeza* reflected accusatory auditory verbal hallucinations and a pathological feeling of guilt. Importantly, these postures did not represent catatonic features or passive experiences. Other than in the two patients described here, these postures and their association with patients' symptoms have sometimes been observed in patients with schizophrenia in clinical practise. We considered these postures to be socially inappropriate and bizarre because *seiza* is usually only performed in traditional Japanese settings, such as during martial arts (*budo*) and tea ceremony (*sado*), and in houses with traditional flooring (*tatami*). Even in Japan, no clinical situation warrants the performance of *seiza*. *Dogeza* is considered to be a comedic position that indicates exaggerated formality, and it is always considered inappropriate and bizarre in a clinical setting.

While we considered these postures to be inappropriate and bizarre, another possibility, especially in Patient 1, is that these postures were contextually appropriate because of the patient's relatively weak position during hospitalisation. Patients are often admitted by force due to their psychotic symptoms and their hospital life is well-organised but limited. Consequently, they tend to feel that they are in a relatively weak position, although we strive to maintain their rights. We partially agree that this can somewhat explain the performance of these postures, but the issue here is the excessive level of formality (Patient 1). Indeed, throughout the unstable clinical course, the excessive formality of Patient 1 was associated with symptom instability rather than restrictions. It was even observed during the initial period of symptom deterioration when restrictions were not yet strict. In addition, he showed an appropriate level of formality when his condition was stable in spite of strict restrictions in the ward. Furthermore, excessive formality seems to be specific to schizophrenia, and it is rarely observed in patients with neurotic disease who can judge the situation and manage their attitude appropriately. Some patients with autism spectrum disorder also show inappropriate attitudes due to difficulty in reading social context, and, as in this case, their inappropriateness is always noticed.

Imbalance between symptoms and social cognition could be involved in the emergence of the *seiza* posture, because expressing formality requires social cognition. The relatively spared social cognition of Patient 1 might lead him to show these postures in the background of schizophrenic symptoms of fear/tension (13) and cognitive impairment including inflexibility, which is difficulty in maintaining an appropriate attitude. In addition, his excessive formality provided us with a new viewpoint for the assessment of schizophrenic symptoms such as social dysfunction. Social cognition impairment is known to occur in patients with schizophrenia (14, 15), and the social attitude of patients with schizophrenia is usually assessed from the viewpoint of difficulty in showing formality. In contrast to reduced formality, excessive formality, as seen in Patient 1, could also be considered social dysfunction, although it may be secondary to schizophrenic symptoms of fear and tension.

In clinical practise, both types of *dogeza* are significantly more out of place than *seiza*. In Patient 1, the entreaty type of *dogeza* was associated with fear and tension (not hallucination or delusion). However, unlike *seiza*, the entreaty type of *dogeza* is generally not considered formal but is rather considered to be comedic and unrealistic. Therefore, *dogeza* may be a more severe sign than s*eiza*. In addition, the apology type of *dogeza* observed in Patient 2 was associated with accusatory auditory hallucination, which is a commonly observed type of schizophrenic auditory hallucination (16). The apology type of *dogeza* is also rarely observed in daily life. It is only observed in life-threatening physical and social situations, which are only encountered in dramas, movies, and possibly in mafia-related situations. We surmise that Patient 2 felt that her auditory hallucination was life-threatening. Indeed, the contents of her auditory hallucinations were life-threatening. In addition, her pathological feeling of guilt may have provoked the performance of *dogeza*. Patients with schizophrenia commonly feel guilty, and one survey demonstrated that female patients showed higher feelings of guilt than male patients (17). Furthermore, Patient 2 showed a posture of *harakiri*, which was a method of committing suicide and expressing sincere apology in Japan in the past that has never been seen in contemporary Japan, indicating her pathological feeling of extreme guilt. Therefore, both the life-threatening accusatory auditory hallucinations and pathological feeling of guilt may have resulted in her performing *dogeza*.

With regards to patients' perspectives, Patient 1 reported that his postures were associated with fear, tension, and anxiety, and not directly associated with hallucinations or delusions. He also reported feeling sorry for the doctor, but he did not provide any reason for this. Patient 2 reported that she found it necessary to apologise as her accusatory hallucinations and delusions resulted in her feeling guilty. It is important to note that both our patients' ability to report their symptoms was severely impaired due to acute psychosis, and clinical assessment based on our knowledge of their postures significantly helped our understanding of their perspectives. Patient 1 reported feeling sorry for the doctor, but his perspective was considered to reflect his fear and tension rather than a pathological feeling of guilt due to accusatory hallucinations, which was the case in Patient 2. Indeed, these perspectives might be difficult to explain because entreaty and apology may be psychologically linked. Apology may include a component of entreaty in that it constitutes a request to accept something that is usually unacceptable (18, 19), and entreaty may include an apology for requesting something unreasonable. Although Patient 2 readily shared her perspective, its deeper understanding, including her extreme pathological feeling of guilt and its association with accusatory hallucinations, required knowledge of these postures.

This case report has several limitations. First, since we have only reported on two patients showing *seiza* and *dogeza* postures, our interpretation cannot be generalised. Other interpretations of these signs cannot be denied, and these signs should be interpreted based on each patients' clinical features and their background. Second, since this was not a systematic research study, quantitative clinical assessment and comparison with control individuals were not performed. Further quantitative research using precise clinical data is required to better understand the significance of these postures in clinical practise. Third, we only reported cases of patients with schizophrenia, and the significance of these postures in other psychiatric disorders remains to be investigated. These postures could potentially be observed in patients with pathological fear, anxiety, or guilt, irrespective of whether they have schizophrenia. However, our clinical experience indicates that these postures are specific to patients with schizophrenia. Further research including medical record surveys is required to reveal the prevalence and specificity/sensitivity of these postures for the diagnosis of schizophrenia.

In conclusion, these culture-dependent postures provided us with plenty of information regarding our patients' clinical states, including schizophrenia-related fear/tension, accusatory auditory verbal hallucinations, and pathological guilt. These inappropriate and bizarre postures initially seemed to reflect social cognition impairment, but careful assessment revealed that this was not the case. Our patients' schizophrenic symptoms resulted in secondary social cognition impairment. Frequent observation of *seiza* in a clinical setting, where it is not needed, may reflect increased fear and tension. *Dogeza* should always be considered abnormal, both in the clinical setting and in the community, and it indicates a severe clinical condition. Asking patients regarding their perspective may help to understand the meaning of their postures, which may include prayer, respect, entreaty, and apology. We believe that the cross-cultural understanding of psychiatric symptoms will extend the frontiers of knowledge in psychiatry, which is important in the current globalised society. We hope our findings inspire psychiatrists of other cultures to investigate similar findings in their patients.

## Data Availability Statement

The raw data supporting the conclusions of this article will be made available by the authors, without undue reservation.

## Ethics Statement

Ethical review and approval was not required for the study on human participants in accordance with the local legislation and institutional requirements. The patients/participants provided their written informed consent to participate in this case report.

## Author Contributions

AK treated the patients and discussed their postures with TK and KO. MO managed the treatment setup. AK wrote the first draught and TK, KO, and MO confirmed it. All authors contributed to the article and approved the submitted version.

## Conflict of Interest

The authors declare that the research was conducted in the absence of any commercial or financial relationships that could be construed as a potential conflict of interest.

## Publisher's Note

All claims expressed in this article are solely those of the authors and do not necessarily represent those of their affiliated organizations, or those of the publisher, the editors and the reviewers. Any product that may be evaluated in this article, or claim that may be made by its manufacturer, is not guaranteed or endorsed by the publisher.
